# Forethought

**Published:** 1869-09

**Authors:** 


### HALL'S JOURNAL OF HEALTH.

OUR LEGITIMATE SCOPE IS ALMOST BOUNDLESS: FOR WHATEVER BEGETS PLEASURABLE AND HARMLESS FEELINGS, PROMOTES HEALTH; AND WHATEVER INDUCES DISAGREEABLE SENSATIONS, ENGENDERS DISEASE.

*We aim to show how Disease may be avoided, and that it is best, when sickness comes, to take no Medicine without consulting an educated Physician.*

VOL. XVI. SEPTEMBER, 1869. [No. 9.

### FORETHOUGHT.

To have one's wits about him under all contingencies, is one of the most valuable practical qualities which a man can possess. It belongs to a strong mind, whether in man or woman; and would save thousands of lives and incalculable suffering every year. One of the means by which we can arrive at a good share of this valuable characteristic is, to fix in the mind what should be done under certain circumstances. To do this, presupposes intelligence.

If a woman's dress is suddenly enveloped in flames, instead of running to her, or out of the house, speak distinctly and commandingly: "Lie down and roll over!" Meanwhile, rip up the carpet, or drag off a bed-blanket, throw it over the person, and then proceed to wrap her up closely in it; this is a more certain and speedy extinguisher than water, is more accessible, and entirely safe to the person giving aid.

If a man faints away, instead of yelling out like a savage, or running to him to lift him up, lay him at full length on his back on the floor, loosen the clothing, push the crowd away so as to allow the air to reach him, and let him alone. Dashing water over a person in a simple fainting-fit is a barbarity, and soils the clothing unnecessarily. The philosophy of a fainting-fit is, the heart fails to send the proper supply of blood to the brain; if the person is erect, that blood has to be thrown up hill, but if lying down, it has to be projected horizontally—which requires less power, is apparent.

If a person swallows a poison, deliberately or by chance, instead of breaking out in multitudinous and incoherent exclamations, dispatch some one for a doctor; meanwhile, run to the kitchen, get half a glass of water in anything that is handy, put into it a teaspoonful of salt and as much ground mustard, stir it an instant, catch a firm hold of the person's nose, the mouth will soon fly open, then down with the mixture, and in a second or two up will come the poison. This will answer in a larger number of cases than any other. If, by this time, the physician has not arrived, make the patient swallow the white of an egg, followed by a cup of strong coffee (because these nullify a larger number of poisons than any other accessible articles), as antidotes for any remaining in the stomach.

If a limb or other part of the body is severely cut, and the blood comes out by spirts or jerks, *per saltem*, as doctors say, be in a hurry, or the man will be dead in five minutes; there is no time to talk or send for a physician: say nothing, out with your handkerchief, throw it around the limb, tie two ends together, put a stick through them, twist it around, tighter and tighter, until the blood ceases to flow. But stop, it does no good. Why? Because only a severed artery throws blood out in jets, and the arteries get their blood from the heart; hence, to stop the flow, the remedy must be applied between the heart and the wounded spot—in other words, *above* the wound. If a vein had been severed, the blood would have flowed in a regular stream, and slow; and on the other hand, the tie should be applied *below* the wound, or on the other side of the wound from the heart, because the blood in the veins flows towards the heart, and there is no need of such great hurry. But we will not tell too much; there are other journals to write, and we do not intend to place ourself in the same category with that unforethoughted class of clergymen who tell all they know in a few first sermons—consequently, soon run out, and are on the hunt for another place.

### EMERGENCIES.

These crowd upon us sometimes. Most unanticipated conjunctures arise, things we could never even have dreamed of, which bring into play all our stock of *presence of mind*, availability of idea—this is having our thoughts "on call," as men say in Wall street. And this is precisely the difference between a smart man and a fool; between a keen operator and a theoretical philosopher. A man who lends his money at three per cent a year, to be returned to him in an hour's notice, makes more than he who lends by the year at twenty per cent; for, in the latter case, it usually stays lent. A very small stock of ideas on call, which can be made use of at a moment's notice, is worth whole cart-loads of magnificent lumber, which require a month to separate and classify We will try the reader on the spot, and very much do we fear that we will find him a numbskull. "About the middle of July, we found ourselves at five o'clock every morning, peering up among the grape-vines, to get at the measuring-worms which were making sad havoc with the leaves and fruit; in a very few days we slew seven thousand three hundred and thirty, one by one; but frequently, while in this operation, we found our mouth was open as well as our eyes; and the thought occurred that one of these worms might fall down our throat; and, in imagination, we felt the ugly creature squirming about in our stomach; and began to look around for a remedy." In the light of what has been said, does a remedy occur to the reader's mind? The salt and mustard emetic would most certainly oust the horrible tenant in double-quick time.

We frequently find ourselves devising ways and means, in probable emergencies, and in inevitable exposures, for coming off safe and sound. In the afternoon of 21st June, Anno Domini, one thousand eight hundred and fifty-eight, we entered an omnibus at the Astor House. There was not a breath of air, the most moderate locomotion occasioned profuse perspiration. A storm was evidently brewing, of no ordinary character. On looking up Broadway, we were reminded of the white squalls which we had seen and encountered in the West Indian seas; and in an instant it was upon us, and in the next, such a pouring-down rain as is seldom witnessed in New York. We felt as if the chances were equal, that the vehicle would be whirling over and over towards the East River; we concluded in our own mind that we would cling to the bottom, and let it whirl on. The rain continued falling at a furious rate, and we were nearing Union Square, without the least sign of abatement. As we had a single block to walk from the Square, the question was, How should we get home? A torrent of rain on a linen coat, the body bathed in perspiration, and in an exhausted condition—not having eaten a particle for some ten hours—visions of pleurisy, pneumonia, peritoneal inflammation, congestive chill, and other such horrors, rose before us, as unwelcomely as the apparition at Endor before King Saul. It was determined at once that we would "abide by" the omnibus until the sky was emptied. Rather a bore to be riding up and down Broadway, by the hour, without an object, and as hungry as a hyena, too. But that was better than sickness, and pills, and plasters, calomel, asafœtida, and aqua fortis. But just as we reached the Square, the rain suddenly ceased, the sun broke from behind a cloud, the atmosphere was cooled, the glittering rain-drops sparkled on the trees of the Park, and we felt the happiness of a first hour in the country—a happiness of briefest duration, for on reaching home, we found the doors and windows all open, a stranger on the step greeted us with the information:

"The house has been struck!"

Any body hurt?

"No."

"All right."

How monosyllabic, how concise and direct, are our speeches on occasions of great moment! No circumlocution, no halting, nothing indefinite.

On entering, we found the floors flooded with water, and daylight coming from a new quarter—to wit, through the roof; for the tornado had lifted a tall chimney from one side of the building, and dashed it on the other, with a force which snapped into splinters five girders, a foot through, and the water, instead of running off by the spout, as it ought to have done, was making a voyage of discovery through the hole in the roof, as large as all out-doors, and that too, within three days after finishing the usual annual house cleaning; for be it known to our Western readers, that once a year, at least, and sometims twice, the Eastern people wash every floor, and every wall not papered, beginning at the attic or garret, and ending with the cellar. It would be considered discreditable to any housewife to allow an omission.

A little while sooner, a very promiscuous scene was exhibited.

At the moment of the terrible crash, grandmother was in bed, convalescent by the merits or demerits of Homœopathy; but by virtue of the infinitesimals, or the chimney, we don't pretend to say which, she didn't remain in bed long. She had taken the forty-nine thousandth dilution early in the morning, but there was no perceptible effect until the chimney, with a thousand of brick, came through the roof; but between the two, she became exceedingly vigorous, and in night-cap and gown, was tearing around the room, ringing an auction-bell. Wifey, for the first time in her life, was struck dumb—which, by the way, was the most extraordinary part of the whole affair, the oldest inhabitant could not remember any similar occurrence, and we have no idea that there will ever be a recurrence of it; but although she said nothing, she did a great deal—being a person of great industry and energy, she paced the floor, and wrung her hands most diligently. When we first laid eyes on her, she had a pocket-handkerchief, or something of that sort, wiping up two or three hogsheads of water from her newly-laid carpet. But amid the wringing of hands and the auction-bell, the clashing of falling water, and the howling of the wind through the building, our three youngest (the fourth being on a visit) were crouching about the room in the utmost terror. At length, no progress being made towards mending matters, our little Bob, in his fifth year, began to grow impatient, and cried out with unusual energy: "What shall we do?—what shall we do, mother?"

"I don't know, my dear—must pray to the Lord, I suppose."

"Why don't you do it, then—why don't you do it?" And down the little fellow dropped on his knees with the most commendable promptitude, and commenced the only prayer he knew:

"Now I lay me down to sleep—"

In a moment, as it were, the rain ceased, and the sun shone out, and Bob, as he got up, saw our dear little Alice, only three and a half, in a corner of the room on her knees, and called out to her: "You needn't pray, now, Ally. Only when you see the dark cloud coming." How many larger children are there, whose piety, like that of little Bob, is only observable in times of imminent peril! Meanwhile, we have forgotten what we began to write about but it is worth while, as of practical inte rest, to add a word about that auction-bell: "Grandmother" being a "Friend," eschews all weapons of war, but has a great horror of burglars; so instead of a Colt's revolver, she has within easy reach, every night, a tremendous auction-bell, which, if rung out of a second-story window at midnight, would be more efficient in attracting attention and routing invaders, than a dozen guns; and we rather think she is right. And in the emergency of the tornado, she naturally thought that something ought to be done anyhow, and the ringing of an auction-bell was as practicable and convenient as anything else; so she rang away. After all, we don't think it at all extraordinary that poor little Ally should go to prayers, for such an odd commingling of sounds was quite enough to confuse and frighten a child of three years.

As to the determination not to leave the omnibus, it was certainly well taken; for a few days later, under very similar circumstances, a widely-known editor got wet to the skin—it threw him into a congestive chill, from the effects of which he died within a week, in the very prime of life.

